# Meeting of the Alumni Association of the Buffalo Medical College

**Published:** 1876-02

**Authors:** 


### Editorial.

### Meeting of the Alumni Association of the Buffalo Medical College.

The committee in charge of the arrangements for the coming meeting of the Alumni Association have so far completed their plans that we can promise for those who attend, a meeting full of interest. All the gentlemen who have been requested to take part in the meeting have signified their intention to be present, and fill their portion of the programme. It is hoped, and from the replies which have been received to the circulars issued, may be confidently expected, that the attendance will be largely in excess of that of last year.

The Medical Department of the University of Buffalo has just cause to be be proud of her record in the past, and all of her graduates, who have the interests of the profession truly at heart, should come forward and show that they will stand by their *Alma Mater* in the efforts which she may make in the future to advance her standard. There can be certainly but few, if any, of her Alumni who do not cherish toward her a sincere love and veneration. Toward those few she cherishes nothing in her heart but pity, that they have wandered from the paths of honesty and uprightness of purpose, and allowed themselves to be warped and twisted out of all semblance of what the true and honest physician should be.

Toward those of her sons who have kept the faith, who have held the name and fame of *Alma Mater* in honest veneration and respect, she reaches out her hands with a hearty welcome home, or an earnest "God bless you," as they continue on their journey.

:o:

Owing to an unavoidable delay in getting matter into type, we are enabled to give our readers a brief account of the Alumni meeting and Commencement exercises.

The Alumni Association was called to order at ten A. M., February 23, by Dr. T. D. Strong, the retiring President, who introduced Prof. J. F. Miner, by whom the Association was welcomed in the following words:

*Mr. President and Gentlemen of the Alumni Association:*

I come in behalf of the University of Buffalo, my colleages and myself, to welcome you to our beautiful and growing city, to these old familiar college halls whose memories of early struggles are never to be forgotten; to the warm greetings of friends and renewal of friendships which have grown stronger by time and change. I have come to welcome you to our firesides, our homes, and our hearts; to extend to you the most cordial and hearty welcome.

We greet you as our faithful and honored sons, as our pride, as our chief joy.

We exultingly point to you as our trophies; memomorials of our victories; monuments of our highest achievements, and say, "Ye are our Laurels." Your individual records of professional attainment and progress combined, will comprise, in a great degree, the history of your *Alma Mater*, which has so signally honored itself in honoring you.

If the Buffalo Medical College takes pride in her honored and distinguished sons, is it not also true that to-day her thousand sons rise up and call her "blessed?" She is blessed in a constant and faithful progeny, whose annual pilgrimages to her honored shrine bespeak fraternal and mutual regard. Long may you cherish for each other the deepest sentiments of friendship and respect. Year after year, as you gather and receive fresh baptism from her pure springs, may you return again to your distant homes, more than ever to appreciate the value of professional honor, and the joys which spring from high aims and pure purposes.

May the day never dawn when your *Alma Mater* shall not exult in your prosperity, taking pride in your achievements. And, oh, may the sun hide itself ere any of the children of this institution would detract from the unspotted character, the fame and fair name of the Buffalo Medical College.

Again, I welcome you to the festivities of this occasion, congratulating you upon the banquet you have spread, the honored and distinguished names you have recorded, the importance and influence of the measures you propose. Wishing you all in your future lives the prosperity and success you so richly deserve, on this occasion a pleasant and profitable social reunion, and a safe return to your various homes, I bid you welcome, thrice welcome.

At the conclusion of Dr. Miner's remarks, Dr. W. W. Jones, the President elect, was introduced, and took the Chair.

The morning session was occupied in reading reports, the election of officers, and in listening to the President's address.

Dr. Jones, in his address, chose the topic of medical education. He spoke of the more prominent faults of our system, but at the same time was not blind to its many good features. He recommended, as the foundation to an improved method, that physicians exercise more discretion in choosing the students whom they admit to their offices. The address was an able and well-considered production.

The Executive Committee having recommended that a triennial prize of $109 be offered by the Association for the best essay on some original research in any of the departments of medicine or surgery, the following gentlemen agreed to be responsible for the amount: Drs. H. D. Vosburg, T. D. Strong, Wm. B. Gould, D. D. Loop, W. W. Potter, E. N. Brush, H. P. Hall, W. W. Jones, C. C. Wyckoff and S. W. Wetmore.

The President appointed as a Committee on Prize Essays, Drs. H. D. Vosburg, T. D. Strong, Wm. B. Gould and E. N. Brush. The above committee organized with Dr. H. D. Vosburg as Chairman, and Dr. E. N. Brush, Secretary.

The essays competing for the prize must be handed in to the Secretary, No. 8 South Division street, Buffalo, N. Y., prior to January 1st, 1877, accompanied by a sealed envelope bearing a motto similar to that on the essay, in which is the name of the writer.

The officers for the ensuing year are as follows :

President—William W. Potter, Mt. Morris, N. Y.
1st Vice-President—Eugene Smith, Detroit, Mich.
2d Vice-President—W. P. Gould Lockport, N. Y.
3d Vice-President—J. W. Craig, Churchville, N. Y.
4th Vice-President—P. H. Clark, Ashland, O.
5th Vice-President—H. Nichell, Buffalo.
Secretary—W. C. Phelps, Buffalo.
Treasurer—Dr. C. C. Wyckoff, Buffalo.
Trustee—C. Diehl, Buffalo.
Executive Committee—W. W. Potter, *ex officio;* M. G. Potter, *ex officio;* A. H. Briggs, Buffalo; E. L. Shurley, Detroit; E. N. Brush, Buffalo.

In the afternoon session, Dr. C. H. Richmond, of Livonia Station, N. Y., read a paper on questions relating to sanitary science. In his paper Dr. Richmond dwelt upon the etiology of Elephantiasis Grecorum and Arabum, Endemic Hæmaturia and Chyluria; and spoke of the means which should be adopted for their prevention.

This paper was followed by a humorous poem by Dr. Fowler Brodnack, of New York, after which Dr. S. H. Benton, of Oil City, Pa., read a paper on Public Hygiene. At the conclusion of Dr. Benton's paper, a somewhat extended discussion was had, in which Drs. White, Hunt, Jones, Richmond, and others, took an active part.

The Commencement exercises at the Hall took place at 7.30 P. M.

The degree of Doctor of Medicine was conferred upon the following graduates :

John Grove Van Pelt, Williamsville; William J. Falkner, St. Catharines, Ont.; Arthur Benedict, Tonawanda; Ben. C. Wakely, Battle Creek; Frederick Lemuel June, Waterport; Chas. Henry Wetzel, Buffalo; Willis Barnard Gifford, Lee, Mass.; Walter D. Greene, Buffalo; Isaac A. M. Dyke, Belmont; Mrs. Mary B. Moody, Buffalo; Plympton Ayres Walling, Corry, Pa.; Charles Ambrose Young, Clarence; John E. Bradshaw, Rose; Arold Albert Freeman, Ripley; John George Miller, Strykersville; George Peter Richardson, Royalton; Wm. Jasper Packwood, Buffalo.; Walter R. Francis, Westfield, Pa.; Chas. Redway Dryer, Victor; Daniel Francis Everts, Farmer Village; Adam Bryan, Benzette, Pa.; John Louis Chewett Cronyn, Buffalo; Edward Austin Adams, A. B., Oakham, Mass; Alvin Allace Hubbell, Leon; Burton Hill Putnam, Westfield; Fred. Eugene King, Trumansburg; Victor Albert Ellsworth, East Otto; Wm. Victor Miller, Buffalo; Wm. James Quinlan, Dunkirk; Chas. Pomeroy Graves, Westfield; Chas. Benj. Brown, Westfield; Joseph R. Laine, Grand Island, Neb.; Heyme Maddelage Werneke, Buffalo; Chas. Osmyn Chester, Buffalo; H. D. Vosburg, Lyons, honoraay degree.

The following prizes were also awarded :

The Fillmore prize, the first of $30 to C. R. Dryer, for the best thesis; the second prize of $20 to A. A. Hubbell.

The prize for the best examination in Anatomy, to B. H. Putnam, of a *post mortem* case.

The prize for tne best report of Prof. White's lectures on the forceps, to Mr. Minges—a complete obstetric case.

The prize for the best report of Prof. Rochester's clinical and didactic lectures on heart diseases, to Charles D. Shepard—a complete stethoscopic case.

Prof. Stodard's prize on Materia Medica, to C. R. Dryer.

Prof. Miner's prize for the best report of his clinical lectures on Surgery, to Mrs. Mary Moody—a Miner pocket case.

Prof. Moore's prize for the best examination in Surgery, to B. H. Putnam,

The address to the graduating class by M. B. Andesrson, LL. D., President of the Rochester University, was a noble and scholarly exhortation to honesty of purpose and purity of life. It was listened to with admiration by all, and was somewhat out of the usual line of addresses on similar occasions. We regret that its being extempore prevents us from giving it in full.

Dr. Anderson was followed by Dr. Sanford B. Hunt, who gave an interesting and instructive address to the Alumni, holding up to them some of the founders of the Bufialo Medical College as noble examples worthy of emulation.

At the conclusion of Dr. Hunt's remarks the benediction was pronounced, and the Alumni Association adjourned to the Tifft House, where a richly-ladened table awaited their coming. After the inner man was satisfied, toasts were in order, and the time was spent in speeches and songs.

The occasion was a very interesting one, and the large number present and the active interest manifested, shows that the Alumni of Buffalo Medical College do not forget their *Alma Mater*.

